# Familial Mediterranean Fever Complicated By Massive Cardiac Tamponade

**DOI:** 10.7759/cureus.50137

**Published:** 2023-12-07

**Authors:** Lara Melo, Haris Patail, Garima Gautam, Julie Braish, David Ozimek

**Affiliations:** 1 Internal Medicine, University of Connecticut Health, Farmington, USA; 2 Leukemia, MD Anderson Cancer Center, Houston, USA

**Keywords:** pericardial effusion, cardiac tamponade, transthoracic echocardiogram(tte), computerized tomography, electrocardiogram (ecg/ekg), familial mediterranean fever

## Abstract

Familial Mediterranean fever (FMF) is a hereditary, autosomal recessive auto-inflammatory disorder characterized by recurrent attacks of fever and serositis. While arthritis, pleuritis, peritonitis, and pericarditis are common in FMF, large pericardial effusions with cardiac tamponade as a sequelae of FMF are considered rare.

We report a case of an 83-year-old female with a history of FMF who presented with chest pain. She was found to have acute pericarditis complicated by hemodynamically significant pericardial tamponade that was subsequently treated with an urgent pericardiocentesis followed by colchicine.

## Introduction

Familial Mediterranean fever (FMF) is an inherited auto-inflammatory disorder following an autosomal recessive pattern [1]. It is known for its distinctive features of recurrent fever accompanied by serositis [1]. Typically emerging in childhood, these episodes last 6 hours to 4 days and resolve spontaneously [2].

FMF is more prevalent in individuals of Mediterranean and Middle Eastern descent [2]. The diagnosis often hinges on the classic presentation of recurrent fever and peritonitis, pleuritis, pericarditis, or arthritis, with symptomatic relief after colchicine treatment [2]. We described a case of FMF of a patient who initially presented with chest pain and was ultimately diagnosed with acute pericarditis, leading to pericardial effusion and pericardial tamponade, a rare yet life-threatening manifestation of FMF.

## Case presentation

An 83-year-old female with a past medical history of FMF (not on treatment) and polymyalgia rheumatica on prednisone presented to the emergency department with a one-day history of fever and abdominal pain. Vital signs were temperature 38.6°C (101.4°F), heart rate 102 beats per minute, respiratory rate 20 breaths per minute, blood pressure 130/97 mmHg, and oxygen saturation 99% on room air. Physical examination revealed a well-kept, alert, and oriented elderly female. Cardiovascular and pulmonary exams were within normal limits aside from mild tachycardia. White blood cell count was 20.7 x 10^3^/uL, hemoglobin 11.1 g/dL, c-reactive protein (CRP) 433, and erythrocyte sedimentation rate (ESR) >130. An electrocardiogram (ECG) showed sinus tachycardia with a chronic right bundle branch block. Her urinalysis was positive for infection and, therefore, she was admitted to the hospital for the management of a urinary tract infection with ceftriaxone. Computerized tomography (CT) of the abdomen was done to evaluate for pyelonephritis; however, it revealed mild enhancement of the pericardium. Routine transthoracic echocardiogram (TTE) was ordered for further evaluation. 

**Figure 1:**
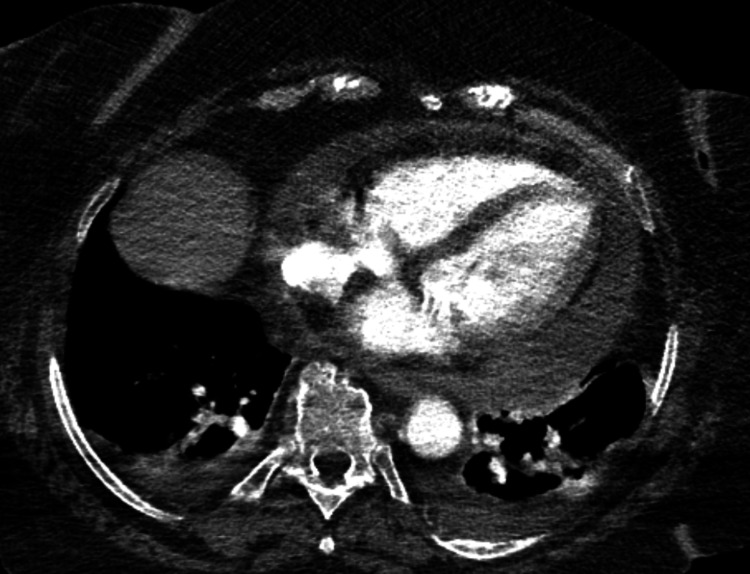
CT chest with contrast showing a large circumferential pericardial effusion CT, computerized tomography.

Despite only having symptoms of fever and abdominal pain on admission, within 24 hours she became altered, confused, tachycardic, and hypotensive. Repeat ECG showed sinus tachycardia, right bundle branch block, and low-voltage QRS. She subsequently underwent a CT chest angiogram, which ruled out pulmonary embolism, but revealed cardiomegaly with a moderate-to-large pericardial effusion (Figure 1).

**Figure 2:**
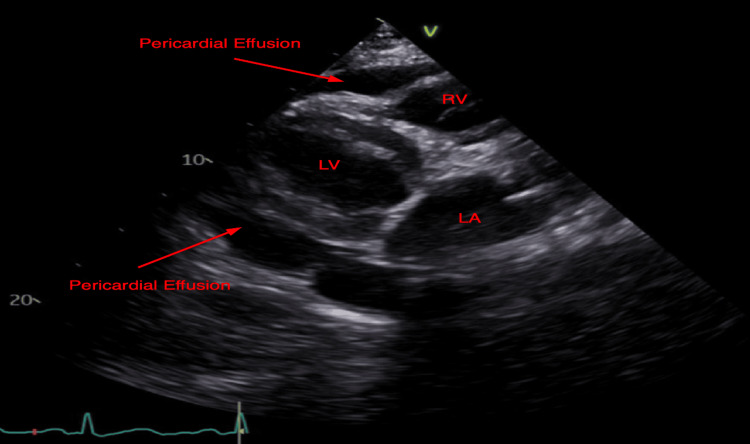
Parasternal long-axis (PSLA) echocardiogram - Moderate-to-large circumferential pericardial effusion with right ventricular collapse RV, right ventricle; LV, left ventricle; LA, left atrium.

**Figure 3:**
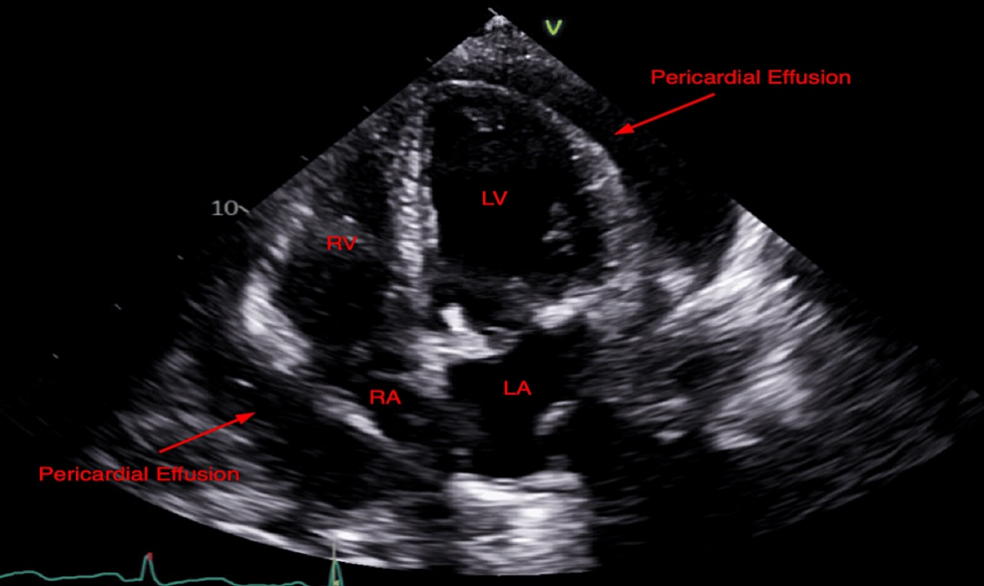
Apical four-chamber echocardiogram - Moderate-to-large circumferential pericardial effusion with right atrial invagination RA, right atrium; RV, right ventricle; LA, left atrium; LV, left ventricle.

**Figure 4:**
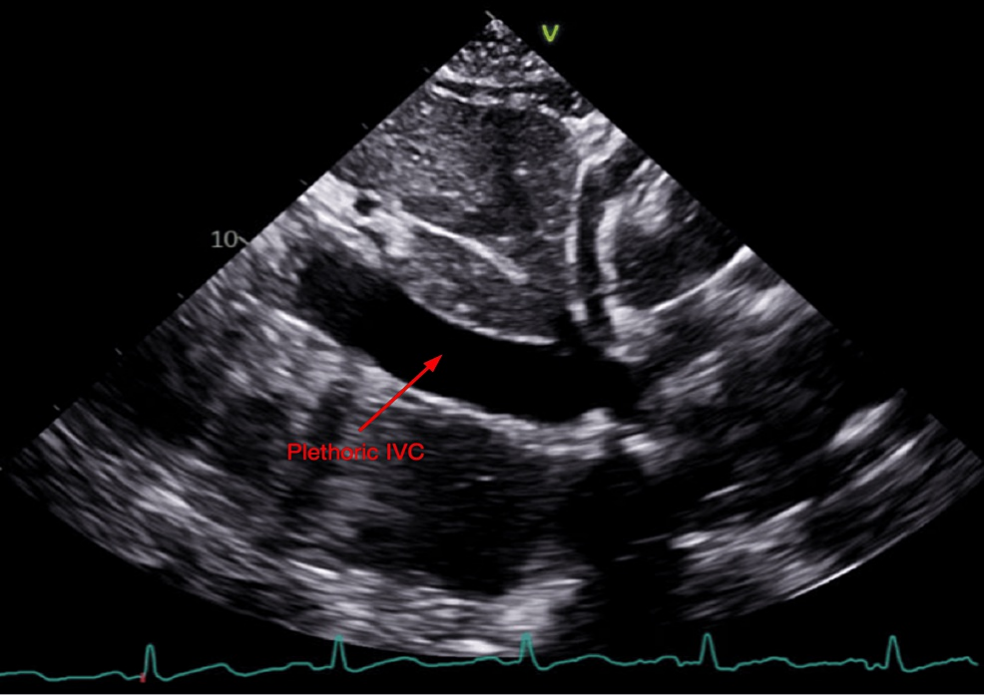
Subcostal IVC echocardiogram - Plethoric IVC IVC, inferior vena cava.

Urgent TTE demonstrated a moderate-to-large pericardial effusion with right ventricular collapse, right atrial invagination, and plethoric inferior vena cava, indicative of cardiac tamponade with hemodynamic compromise (Figures 2-4). She was transferred to the medical intensive care unit (MICU) and underwent urgent pericardiocentesis. A moderate hemorrhagic pericardial effusion with loculations was noted. The pericardial fluid had a large number of neutrophils consistent with acute pericarditis and negative for other pathologies. Two drains were placed within the pericardial space and the patient returned to the MICU hemodynamically stable, intubated, and mechanically ventilated. She was started on colchicine, stress dose steroids, and was extubated the next day. Her pericardial drain had minimal sanguineous output and was removed a few days after surgery.

## Discussion

FMF is an autosomal recessive disease, and its epidemiology is predominantly noted within the Mediterranean region [1,2]. It is most prevalent in individuals of Armenian, North African, Jewish, Turkish, and Arab descent. In the United States, FMF is frequently encountered in Ashkenazi Jews and people from the Middle East and Armenia [2].

Affected individuals have pathogenic mutations in the *MEFV* gene located on the short arm of chromosome 16 [2]. The MEFV gene encodes pyrin, a protein that is expressed predominantly in cells of myeloid lineage along with synovial fibroblasts and dendritic cells [2]. Pathogenic mutations in the MEFV gene cause a gain of function in the pyrin protein, leading to a cascade of inflammation, even in the absence of provocation by a toxin or infection, resulting in FMF attack [3,4]. The disease has a variable penetrance, possibly due to other genetic and environmental factors that influence the clinical features and cause differences in disease manifestations [5,6].

The diagnosis should be suspected in individuals with recurrent febrile episodes accompanied by peritonitis, synovitis or pleuritis, recurrent erysipelas like erythema, repeated laparotomies for an acute abdomen with no identifiable underlying pathology, a first-degree relative with FMF, and/or membership in an at-risk ethnic group. Genetic testing is used to further confirm the diagnosis of FMF and to exclude other auto-inflammatory syndromes mimicking FMF. In 1997, conservative diagnostic criteria were created, which included the presence of one major or two minor criteria, or one minor plus five supportive criteria, with a sensitivity and specificity of >95% and >97%, respectively [7]. Major criteria include “typical” attacks of pleuritis, peritonitis, pericarditis, arthritis, and fever, while minor attacks include “incomplete” attacks within the abdomen, chest, or joints, exertional lower extremity pain, or resolution of symptoms with colchicine therapy [6]. Supportive criteria further include a family history of FMF, association with ethnic origin, severity of attacks and duration of symptom-free periods, age of onset (<20 years of age), episodic proteinuria/hematuria, history of laparotomy or white appendix, and parental consanguinity [6]. 

As FMF is characterized by recurrent abrupt attacks of fever, joint pain, and bouts of serositis, episodes are thought to usually last for one to three days and resolve spontaneously [1,2]. Rare manifestations include acute pericarditis, acute scrotum, and protracted febrile myalgia [8]. Cardiac involvement does not typically manifest as the first presentation of FMF; however, there are case reports of adolescents who present with either pericarditis or pericardial effusion [9]. Pericarditis has traditionally been considered a rare complication [8]; however, more recently, it is considered a more classic sequel of FMF [10]. Pericardial involvement is thought to develop in 2.4% of patients with FMF, with infrequent recurrent attacks and rare evolution to pericardial tamponade or constrictive pericarditis [2]. When managed with both colchicine and prednisone, patients usually improve after sustaining an FMF flare with pericarditis [8].

Despite pericarditis becoming more known as a complication of FMF, large pericardial effusions leading to hemodynamic compromise are still considered rare [9]. Within a comprehensive review of FMF in 2021, Alsara et al. noted five different cases of cardiac tamponade within their observed literature review [10]. Furthermore, in 2022, Malek et al. described a case of a 13-year-old boy who presented with pericardial effusion and was diagnosed with FMF for the first time [9]. Continued vigilance and expanded research are needed to comprehend FMF's cardiac manifestations better.

## Conclusions

Despite the rare occurrence of cardiac manifestations related to FMF, providers should be aware of large pericardial effusions and cardiac tamponade as potential complications of FMF. Early diagnosis with work-up, including ECG, CT chest imaging, and quick echocardiogram turn-around times, may provide more insight into patient care. 

Internists and cardiologists alike should remain aware of the rare possibility of avoiding life-threatening hemodynamic changes in patients who have an existing diagnosis of FMF or are presumed high-risk.
